# Prioritizing protein complexes implicated in human diseases by network optimization

**DOI:** 10.1186/1752-0509-8-S1-S2

**Published:** 2014-01-24

**Authors:** Yong Chen, Thibault Jacquemin, Shuyan Zhang, Rui Jiang

**Affiliations:** 1 School of Information Science and Engineering, University of Jinan, Jinan 250014, China; 2 MOE Key Laboratory of Bioinformatics and Bioinformatics Division, TNLIST/Department of Automation, Tsinghua University, Beijing 100084, China; 3 Institute of Biophysics, Chinese Academy of Sciences, Beijing 100101, China

**Keywords:** Complex Disease, Protein Complex, Genomic Data Integration, Network Optimization

## Abstract

**Background:**

The detection of associations between protein complexes and human inherited diseases is of great importance in understanding mechanisms of diseases. Dysfunctions of a protein complex are usually defined by its member disturbance and consequently result in certain diseases. Although individual disease proteins have been widely predicted, computational methods are still absent for systematically investigating disease-related protein complexes.

**Results:**

We propose a method, MAXCOM, for the prioritization of candidate protein complexes. MAXCOM performs a maximum information flow algorithm to optimize relationships between a query disease and candidate protein complexes through a heterogeneous network that is constructed by combining protein-protein interactions and disease phenotypic similarities. Cross-validation experiments on 539 protein complexes show that MAXCOM can rank 382 (70.87%) protein complexes at the top against protein complexes constructed at random. Permutation experiments further confirm that MAXCOM is robust to the network structure and parameters involved. We further analyze protein complexes ranked among top ten for breast cancer and demonstrate that the SWI/SNF complex is potentially associated with breast cancer.

**Conclusions:**

MAXCOM is an effective method for the discovery of disease-related protein complexes based on network optimization. The high performance and robustness of this approach can facilitate not only pathologic studies of diseases, but also the design of drugs targeting on multiple proteins.

## Background

Protein complexes are essential cellular functional units in which several proteins work as parts of assemblies. The functionality of a protein complex is based on interactions of its member proteins that are typically densely connected in a protein-protein interaction (PPI) network, reflecting the modular organization of the network. In pathogenic conditions, dysfunctions of complex members usually affect the entire function of the complex [1-3]. Although systematic genetic and epigenetic analyses in human inherited diseases have revealed numerous SNPs [4-9], miRNAs [10], long noncoding RNAs [11], individual disease proteins [12] and epigenetic modifications [13], functional associations between diseases and protein complexes are still lack of systematic investigations.

Protein complexes have been experimentally and computationally proved to be associated with amounts of diseases. For example, different mutations in SWI/SNF chromatin remodelling complex were reported to cause Coffin-Siris syndrome [14,15], Nicolaides-Baraitser syndrome [16], and cancers [17,18]. Aberration in mitochondrial complex-I NADH dehydrogenase activity could profoundly enhance the aggressiveness of human breast cancer cells, while therapeutic normalization of the NAD+/NADH balance could inhibit metastasis and prevent disease progression [19]. mTOR complex 1 played a critical role in hematopoiesis and Pten-loss-evoked leukemogenesis [20]. In recent years, several system-level maps of protein complexes have been constructed in yeast [21-23], drosophila melnogaster [24] and human [25], presenting significant efforts towards comprehensive understanding of protein complexes. Effective utilization of these large-scale data has been validated useful in analyzing individual disease proteins or related complexes. For example, Lage et al. prioritized disease proteins based on a systematic analysis of human protein complexes comprising gene products implicated in many different categories of human disease [26]. Vanunu et al. provided a global network-based method for prioritizing disease proteins and inferring protein complex associations with a disease of interest [27]. Yang et al. proposed a technique for predicting disease proteins based on a constructed protein complex network [28]. Although these studies, together with early studies of individual disease proteins [29-36], have achieved remarkable successes, large-scale predictions and mechanistic explanations of disease-related complex still remain an open question. Considering that functional units are often protein complexes rather than individual proteins, we highlight the perspective of disease-related complexes rather than disease-related proteins to obtain an up-level investigation that may be one step closer to biological reality.

To this aim, we propose in this paper a computational method, MAXCOM, to prioritize candidate protein complexes. To optimize the relationship between a query disease and a protein complex, the maximum information flow (MIF) between them is calculated through a heterogeneous network that is constructed by using protein-protein interactions and disease phenotypic similarities. MAXCOM then prioritizes all candidate complexes by ranking the MIFs of them. We test, in a cross-validation setting, the utility of MAXCOM in prioritizing protein complex with at least one known gene. Results show that MAXCOM can recall higher proportion of complexes at top one against large randomly constructed negative controls. We also demonstrate the power of MAXCOM by studying the associations of breast cancer and SWI/SNF complex. We believe that our method and predictions provide a useful platform for initially investigating how protein complexes link their actions to development and homeostasis of human diseases.

## Materials and methods

### Workflow of MAXCOM

**Figure 1:**
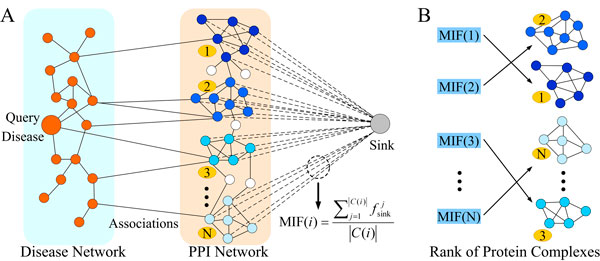
**Workflow of MAXCOM**. A. A heterogeneous network is constructed by combining disease similarity network, disease-gene associations and protein-protein interaction network (PPI). For a query disease and a set of candidate protein complexes, MAXCOM applies a maximum flow algorithm to calculate the maximum information flow (MIF) from the query to each complex. MIF of *i*-th complex is defined as $MIF\left(i\right)=\sum_{j=1}^{\left|C\left(i\right)\right|}f_{\text{ sink}}^{j}/\left|C\left(i\right)\right|$, where $\left|C\left(i\right)\right|$ is the protein number of complex C(i) and $f_{\text{sink}}^{j}$ is the flow value of *j*-th edge from *j*-th protein to sink node. B. Candidate complexes are ranked by the MIFs.

The prioritization of protein complexes is modelled as an optimization problem, in which the objective is to find the maximum information flow between a query disease and a candidate complex through a heterogeneous network. MAXCOM takes several steps to prioritize all candidate complexes to a query disease (Figure 1). First, a heterogeneous network is constructed by the disease phenotypic similarities, disease-gene associations and PPI interactions. Nodes of the network are defined as either diseases or proteins, while the capacities of edges are weighted as the phenotypic similarities among diseases or interactions among proteins. Second, in order to describe the relationship of a query disease and a protein complex, we add an extra sink with edges linked from each members of the complex to the sink. Third, after calculating the maximum information flow from the query disease to this sink, we obtain the maximum information flow (MIF) from the query disease through the nodes of a complex (Figure 1A). For all candidate protein complexes, maximum information flows are calculated, and the complexes are then ranked (Figure 1B). In the following parts, we describe the construction of heterogeneous network and the calculation of maximum information flows of candidate complexes.

### Construction of heterogeneous network

The heterogeneous network is composed of disease phenotypic similarities, disease-protein associations and protein-protein interactions. The phenotypic similarities were downloaded from the literature [37], including pairwise similarities for 5,080 disease. The similarity is ranged from 0 to 1, where a larger value means higher phenotypic similar between a disease pair and vice versa. The PPI network was extracted from the Human Protein Reference Database (HPRD, released in February 2013) [38], including 9,998 proteins and 41,049 interactions. The disease-protein associations were extracted from the Ensemble database by using the Biomart tool [39]. Focusing on the 5,080 diseases and proteins that can be mapped back to the HPRD database, we obtain a total of 1,962 associations between 1,548 diseases and 1,244 proteins. When constructing the heterogeneous network, all the 5,080 diseases and 9,998 proteins are taken as nodes. Edges are composed of the 41,049 interactions between proteins, the 1,962 disease-protein associations and the edges of disease pairs with nonzero similarities. To filter the small similarities that mean low confidences among disease pairs, we introduce a parameter to remove the edges that similarities are less than α=0.1, the mean of all disease similarities. Existing studies have shown that relationships between diseases have noises [37], and thus a noise filtering process is helpful in improving the performance of detecting disease genes [33]. Finally, we obtain a heterogeneous network including 15,078 nodes and to 5,782,818 edges.

To optimize the relationship of a query disease and a complex, we modelled it as the MIF from the query disease node to the sink through all member proteins of the complex (Figure 1A). Here the heterogeneous network is served as a functional network that link diseases and proteins. The MIF is served to measure the value of functional relationship between a query disease and a candidate complex. Intuitively, if the query disease has stronger functional relationship to a candidate complex, the MIF between the disease and the complex will be larger than those the disease to other candidate complexes. For this modelling, a capacity that means the upper bound of connecting information flow is assigned to each edge of the heterogeneous network. In detail, the capacities of edges among diseases are assigned as the same as their phenotypic similarities. The capacities of edges among proteins (protein interactions) are assigned as 1. The capacities of edges among diseases and proteins (disease-protein associations) are assigned as infinite. We also add edges from each protein member of a complex to an additional sink node, and assign the capacities of these edges as infinite. By the capacity definition, if two nodes have a stronger functional relationship, the capacity of the edge between them is larger.

### Calculation of maximum information flow

For the heterogeneous network , where , ,  representing the nodes, edge and nonnegative capacity on each edge respectively, the MIF from the query node to the sink through all the proteins of the complex is calculated by two steps. First, the MIF from the query node to the additional sink is calculated as follows.

(1) $$Maximize:f\left(query\right)=\underset{v\in V}{\sum }f\left(query,v\right),$$

$$s.t\;\underset{v,w\in V}{\sum }f\left(v,w\right)-\underset{v,w\in V}{\sum }f\left(w,v\right)=0,$$

f(v,w)≤cap(v,w),

where the information flow f(v,w) is defined as the flow value transmitted from node  to node , and  the capacity of the edge linked nodes  and .

Second, the MIF from the query to *i*-th complex is defined as , where $\left|C\left(i\right)\right|$ is the protein number of complex C(i) and $f_{\text{sink}}^{j}$ is the flow value of *j*-th edge from *j*-th protein to the sink node. We use the HR_PR algorithm [40] to solve the problem (1). For all candidate complexes, the MIFs are then calculated and ranked.

### Validation method and evaluation criteria

Leave-one-out cross-validation experiments are adopted to assess the capability of MAXCOM in identifying protein complexes that are associated with human diseases. For this purpose, we define a protein complex to be associated with a disease if at least one member protein of the complex has been annotated as associated with the disease. After mapping on 5,080 diseases and 9,998 proteins, a total of 539 disease-related protein complexes are collected from the CORUM database (released in February 2013) [41]. In each validation run, a test protein complex (a positive control) is selected and all the associations between the complex and diseases are deleted. The test protein complex is then ranked against a collection of negative control complexes. Two types of negative control complexes are used in each run of validations. First, 99 random protein complexes are collected as random control protein complexes. For each complex, same number proteins with the positive control are randomly selected from 9,998 proteins. Second, for a given protein complex, all the left 538 protein complexes are considered as negative controls that we named as real control protein complexes for convenient.

Three criteria are used to quantify the performances of MAXCOM. First, if a positive control complex is ranked at the top in a validation run, it is considered as a successful prediction. We calculate the top ranked ratio (TOP) as the number of all successful predictions divided by all validation runs. Second, we calculate the average rank of all positive controls and normalize it by the lengths of ranking lists to obtain a mean rank ratio (MRR). Third, given a threshold of the relative rank, we calculate the sensitivity (true positive rate) as the fraction of test protein complexes ranked above the threshold and the specificity (true negative rate) as the fraction of control protein complexes ranked below the threshold. A rank receiver operating characteristic curve (ROC) is then drawn by varying the threshold value from 0 to 1, and the area under this curve (AUC) is calculated. Obviously, larger TOP and AUC, as well as smaller MRR indicate higher performance.

## Results

### Performance of MAXCOM

**Figure 2:**
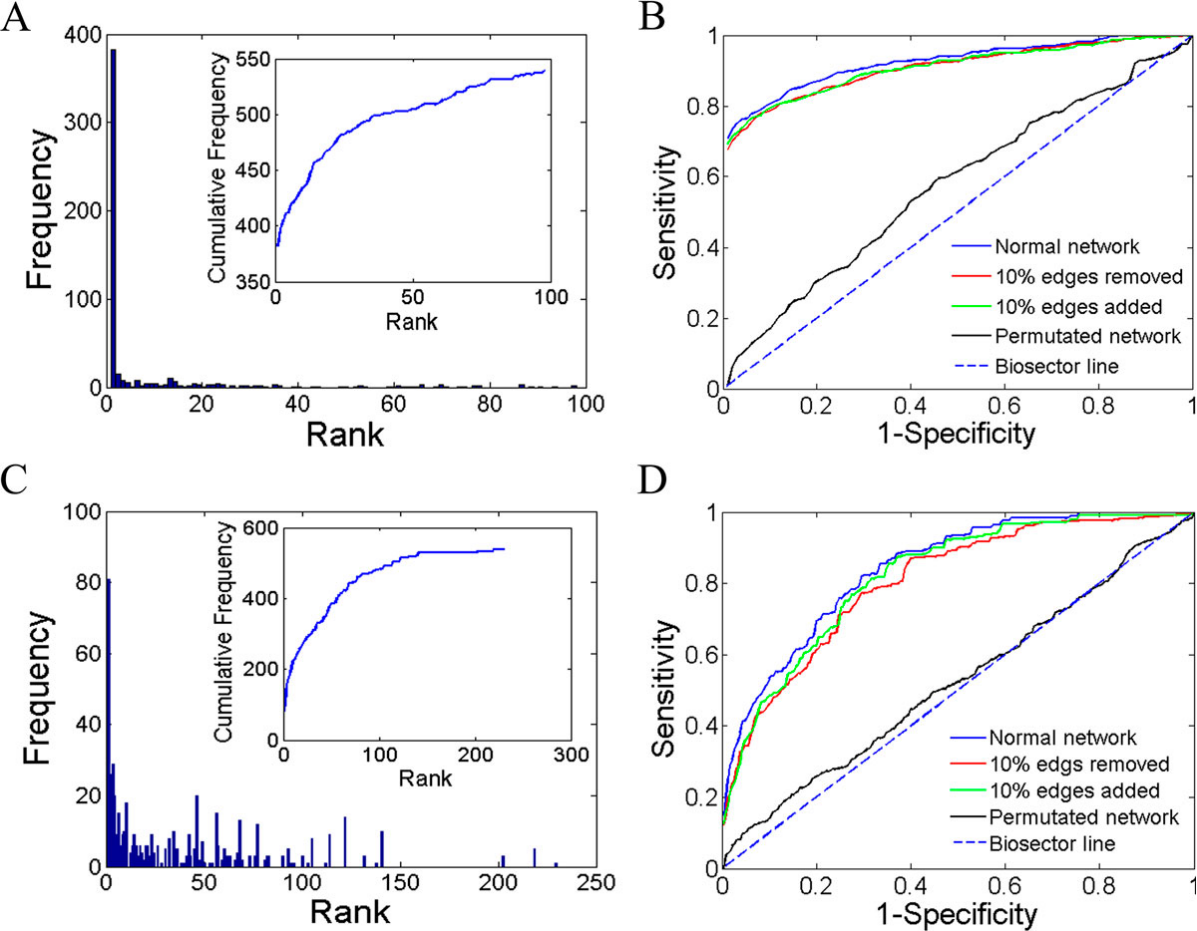
**Performance of MAXCOM**. Histogram of ranks on random control protein complexes (A) and real control protein complexes (C). The rank receiver operating characteristic (ROC) curves on random control protein complexes (B) and real control protein complexes (D). The results were obtained by validating on normal network, 10% deleted or added networks, and randomly permutated network with same node distribution, respectively.

To examine how well MAXCOM prioritizes candidate protein complexes, we assessed its capability of uncovering 539 protein complexes with known disease proteins by using the leave-one-out cross-validation experiments. For each of these protein complexes, we first generated 99 randomly constructed complexes as negative controls. By counting the number of test protein complexes with different ranking positions, we observed that 382 of all 539 test cases are ranked top one, achieving a TOP value of 70.87%. The mean rank ratio (MRR) was only 8.69% and a total of 412 test cases were ranked in top 5, suggesting a faster accumulation of top rankings (Figure 2A). The area (AUC) under the rank receiver operating characteristic curve was calculated as high as 91.33% (Figure 2B).

To simulate the real case in disease studies that user may want pinpoint known complexes for further biological validations, we performed a cross-validation on all 539 disease-related complexes. With a complex selected as positive control, the left 538 complexes were taken as negative controls. In this critical version, MAXCOM also exhibited a faster accumulation of top rankings (Figure 2C). For example, it achieved a TOP value of 15.03, and a high proportion as 30.61% in top 5. Its MRR and AUC were 37.71% and 84.25% (Figure 2D). Although these criteria were all dropped, the decrease was reasonable because the size of negative controls was more than 5.43 (538/99) fold compared that used as random control protein complexes. Thus, MAXCOM also achieved acceptable performances in pinpointing real protein complexes from a set of disease-related complexes and was suitable for large-scale predictions.

### Robustness to network structure

The robustness of MAXCOM in operating potential noise in biological networks is of great important because much noise is widely observed in existing biological data [42,43]. The noise may lead to many negative protein-protein interactions in constructed network and affect the predicting precision. To demonstrate this issue, we employed several strategies to check the robustness of MAXCOM to network structure on both type of control sets. First, we randomly deleted 10% edges of the heterogeneous network. On random control protein complexes, MAXCOM achieved a TOP of 69.02%, an MRR of 10.02% and an AUC of 90.12%. The decreases in these same validation experiments were as small as 1.85% for TOP, 1.33% for MRR and 1.21% for AUC. On real control protein complexes, MAXCOM achieved a TOP of 12.62%, an MRR of 39.92% and an AUC of 80.42%. The decreases in these same validation experiments were as small as 2.41% for TOP, 2.21% for MRR and 3.83% for AUC.

Second, we randomly added 10% edges of the heterogeneous network. At this case, MAXCOM achieved a TOP of 70.5%, an MRR of 9.83% and an AUC of 90.16% on random control protein complexes. The decreases in these same validation experiments were as small as 0.37% for TOP, 1.14% for MRR and 1.17% for AUC. On real control protein complexes, MAXCOM achieved a TOP of 12.8%, an MRR of 38.56% and an AUC of 82.02%. The decreases in these same validation experiments were as small as 2.23% for TOP, 0.85% for MRR and 2.23% for AUC (Figure 2B, D). These two permutation validations suggested that MAXCOM was effective in dealing with false positive edges and shows robustness to network structures.

Third, validation experiments were also performed by shuffling edges in the heterogeneous network but fixing the degree distribution (i.e., the number of neighbours of each node). For this permutated network, the AUC scores were both reduced by approximately 50% on both control sets, while the result for the random control protein complexes was slightly higher as 57.34% (Figure 2B, D). This validation further indicated that MAXCOM could exploit the useful information in the heterogeneous network to prioritize the disease-related protein complexes.

### Robustness to parameter

We also introduced a parameter  to filter out the potential noise of disease similarities. In practice, threshold parameter  played important functions not only in filtering out low confidence values among diseases to improve predicting precisions but also in making the heterogeneous network sparse to speed up running time. Here we changed it with a step as 0.05 to test its effect on MAXCOM (Table 1). If no any disease edges cut off (), the TOP, MRR and AUC were 69.94%, 9.05% and 90.91%, respectively. With the increase of , best performance was achieved at  as we had shown in above paragraphs. With continue increase of , most of criteria came to decrease, especially the TOP. Although these changes were observed, we noticed that changed ratios of three criteria were ranged only very slightly. For example, when  changed from 0.1 to 0.4, the TOP changed from 70.87% to 55.29%, achieving a changed ratio of 21.98%. The MRR changed from 8.69% to 7.46%, and the changed ratio was 14.15%. Meanwhile, the AUC changed from 91.33% to 92.57%, achieving a little changed ratio of 1.36%. These results showed that  was useful to improve the precision of MAXCOM by filtering noise (compared the case of ), and confirmed that MAXCOM was robust to this parameter changing.

**Table 1:** Robustness of MAXCOM with respect to parameter .

	0	0.05	0.1	0.15	0.2	0.25	0.3	0.35	0.4
TOP	69.94%	70.13%	70.87%	69.39%	68.09%	66.23%	64.19%	58.81%	55.29%
MRR	9.05%	8.72%	8.69%	9.09%	9.49%	9.45%	8.34%	8.06%	7.46%
AUC	90.91%	91.14%	91.33%	90.85%	90.45%	90.33%	91.67%	91.78%	92.57%

The parameter  also affected the number of edges in the heterogeneous network. When α=0, there were total 10,174,820 edges in the network. The number was drastically decreased to 5,782,818 (α=0.1) and 154,692 (α=0.4). Thus, with the increase of , MAXCOM ran much faster in calculating. For example, when , the average calculating time of each run was 2.86 seconds. It was dropped to 1.57 and 0.18 seconds when  is 0.1 and 0.4 respectively. For summary,  was useful for filtering low confidence values among diseases and beneficial for improving performances and calculation time of MAXCOM.

### Prediction of protein complexes associated with breast cancer

**Figure 3:**
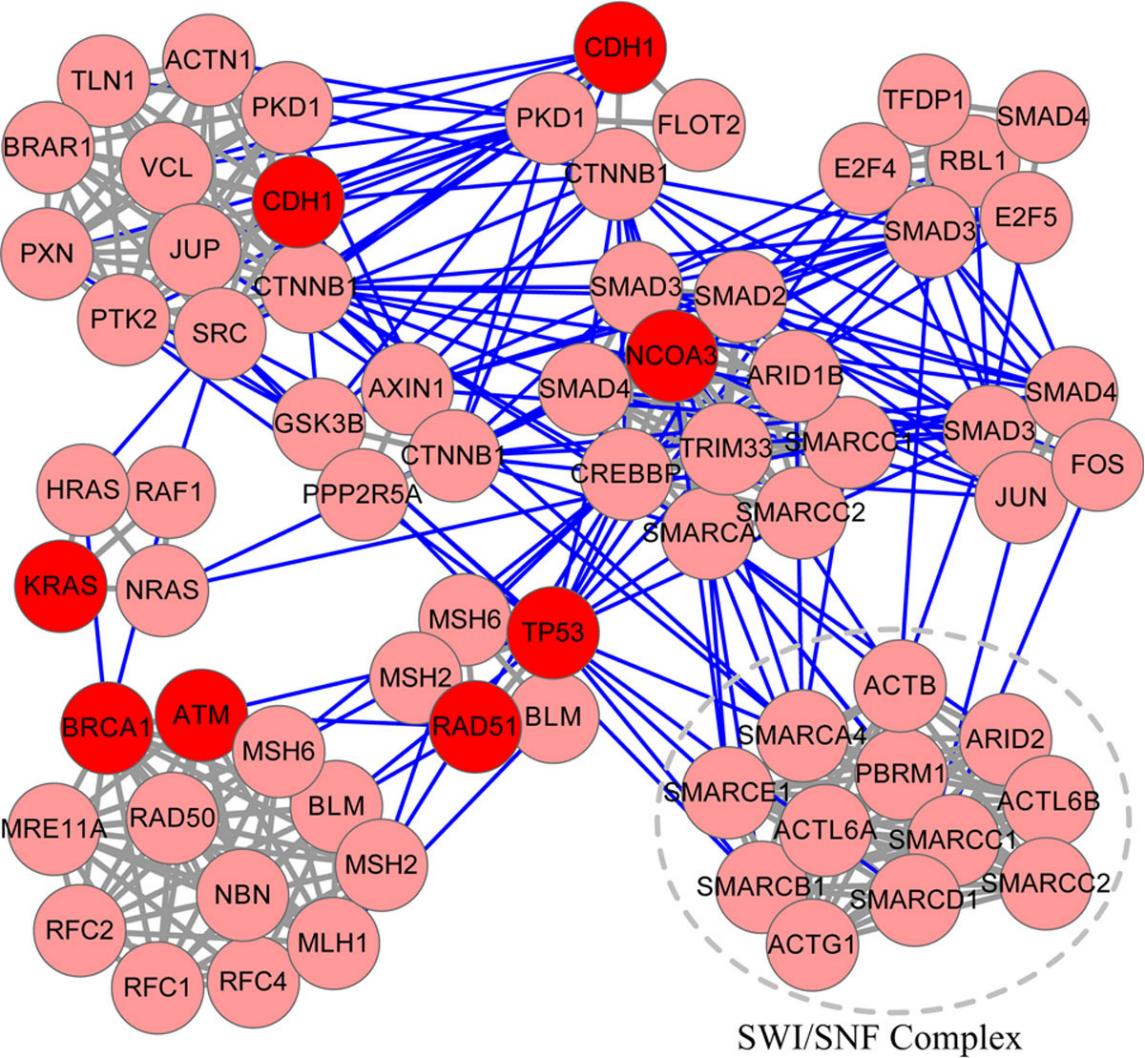
**Interactions of ten predicted protein complexes of breast cancer**. The interactions are shown for 58 proteins of ten complexes. Six known genes associated with breast cancer are noted in red (CDH1, KRAS, BRAC1, ATM, RAD51, TP53). All these ten complexes are connected by protein-protein interactions among them (blue lines).

To demonstrate MAXCOM's ability in predicting novel disease-related complexes, we performed a case study of breast cancer (OMIM 114480), one of the most commonly occurring cancers. We systematically examined the top ten complexes that were prioritized through 539 candidates (Table 2). There were 58 proteins in these ten complexes, including 6 (BRCA1, TP53, KRAS, ATM, CDH1, RAD51) of 32 disease proteins reported in OMIM database [44]. We first preformed a functional enrichment analysis of these 58 proteins by using DAVID database [45,46]. Results showed that these proteins were mostly enriched in chromosome organization (p-value = 1.36e-15), chromatin modification/remodelling/organization (p-value = 7.32e-11) and protein complex biogenesis/assembly (p-value = 9.03e-10). This was consistent with the functional characterizations of the ten protein complexes that were manually annotated by CORUM database [41] (Table 2). Except for known disease proteins of breast cancer that found in the 6 protein complexes, many disease proteins that were associated with many other types of diseases could be found, with examples including E2F4, E2F5, HRAS, JUN, FOS. We also found that proteins (CDH1, CTNNB1, SMAD3, SMAD4, SMARCA4, SMARCC1, SMARCC2) were common in several complexes and all these complexes were connected by amounts of protein-protein interactions (Figure 3), suggesting tight functional relationships among these protein complexes. These results indicated that these complexes might serve as a large functional module involved in different stages of breast cancer.

**Table 2:** Predicted top ten protein complexes of breast cancer.

Complex Name	Entrez ID	Gene Symbol	Functional Characterization
RAF1-RAS complex, EGF induced	3265, 3845, 4893, 5894	HRAS, KRAS, NRAS, RAF1	Enzyme mediated signal transduction
RSmad complex	4087, 4088, 4089, 6597, 6599, 6601, 51592, 8202, 1387, 57492	SMAD2, SMAD3, SMAD4, SMARCA4, SMARCC1, SMARCC2, TRIM33, NCOA3, CREBBP, ARID1B	Transcriptional control; TGF-beta-receptor signalling pathway
Polycysting-1 multiprotein complex	87, 9564, 999, 1499, 3728, 5310, 5747, 5829, 6714, 7094, 7414	ACTN1, BRAR1, CDH1, CTNNB1, JUP, PKD1, PTK2, PXN, SRC, TLN1, VCL	Cell adhesion; epithelium
BASC complex (BRCA1-associated genome surveillance complex)	5981, 5982, 5984, 4292, 4436, 2956, 673, 641, 472, 4361, 4683, 10111	RFC1, RFC2, RFC4, MLH1, MSH2, MSH6, BRCA1, BLM, ATM, MRE11A, NBN, RAD50	DNA repair; DNA damage response
MSH2/6-BLM-p53-RAD51 complex	7157, 4436, 2956, 5888, 641	TP53, MSH2, MSH6, RAD51, BLM	DNA repair; DNA damage response
Polycystin-1-E-cadherin-beta-catenin-Flotillin-2 complex	999, 1499, 2319, 5310	CDH1, CTNNB1, FLOT2, PKD1	Lipid binding; intercellular junction (gap junction/adherens junction); epithelium
SMAD3-SMAD4-cJun-cFos complex	2353, 4088, 4089, 3725	FOS, SMAD3, SMAD4, JUN	Transcription activation; DNA binding; TGF-beta-receptor signalling pathway
SMAD3/4-E2F4/5-p107-DP1 complex	1874, 1875, 5933, 4088, 4089, 7027	E2F4, E2F5, RBL1, SMAD3, SMAD4, TFDP1	Transcription repression; DNA binding TGF-beta-receptor signalling pathway
Axin-PP2A A-PP2A C-GSK3-beta-beta-catenin complex	8312, 1499, 2932, 5525	AXIN1, CTNNB1, GSK3B, PPP2R5A	Wnt signalling pathway
PBAF complex (SWI/SNF complex)	6597, 6598, 6599, 6601, 6602, 6605, 60, 71, 86, 51412, 196528, 55193	SMARCA4, SMARCB1, SMARCC1, SMARCC2, SMARCD1, SMARCE1, ACTB, ACTG1, ACTL6A, ACTL6B, ARID2, PBRM1	DNA conformation modification; transcription activation; DNA binding; hormone mediated signal transduction; ligand-dependent nuclear receptors; organization of chromosome structure

We then analyzed, in detail, the PBAF complex (SWI/SNF complex) since it did not include known disease proteins of breast cancer according to OMIM database (until Aug. 20, 2013) and was listed at last in our ten analyzed complexes. SWI/SNF complex was a multi-subunit chromatin-remodelling complex which mobilizes nucleosomes and remodel chromatin, playing key roles in control of lineage specification, gene expression and repression, metastasis, epigenetic tumor suppression. We found numerous literatures reported that SWI/SNF complex was associated a variety of cancers, including breast cancer. As inactivating mutations in several SWI/SNF subunits had recently been identified at a high frequency in a variety of cancers, a widespread role in tumour suppression had been proposed to SWI/SNF complex [17,47,48]. Actually, SWI/SNF had been demonstrated as the most frequently mutated chromatin-regulatory complex in human cancer, exhibiting a broad mutation pattern, similar to that of TP53 [18]. Here we predicted SWI/SNF in top positions as one of potential protein complexes that were involved in breast cancer. For summary, these proposed ten protein complexes were potentially involved in basic biological functions and agree well with current knowledge on breast cancer.

## Discussion

With the explosion of large-scale "omics" data, computational methods of integrating these complex heterogeneous data can provide a more thorough and systemic analysis for characterizing disease related factors. Here we have proposed a network-based strategy to prioritize candidate protein complexes by integrating disease phenotypic similarities and protein-protein interactions. As analyzed in validation results, MAXCOM is useful in tracing relationships of diseases and complexes through the heterogeneous network. Compared with early works for prioritizing individual disease proteins [12,29,30], our work presents a computational tool to analysis disease related factors at an up functional level and close a step to mechanisms underling diseases.

Although MAXCOM is proved useful, some methodological improvements may be necessary in further research. An important extension is how to describe the tissue specificity. Since different cells have specific cellular functions such as regulation and expression [49], splicing and mehtylation [50], human PPIs and protein complexes in a tissue-specific context have been observed [51]. By utilizing these tissue-specific protein interactions, we may analyze protein complexes towards tissue-specific diseases. Another extension is to consider the "edge prioritization" that suggested in early literatures [12,52]. Instead of only prioritizing proteins or protein complexes in isolation, more attentions should be also devoted to potential interactions among top candidates. Here, we have shown that the top ten ranked protein complexes are functional associated, however a more comprehensive and systematic analysis of these top ranked candidates is desired. In general, this is especially important for following experimental validations, since the correlations of top ranked protein complexes may usually indicate a time and spatial cellular relationships. Third, the noise filtering is another highlight to be addressed. Considering that all the biological data are far from complete and full of noise, it is extremely useful to improve the precision by filtering noise before data integration. There are two different ways that can be used for this aim. The one is to filter low confidence data by parameters as used in our study, the other is by integrating more relevant types of biological information. For example, the relationships among proteins can be described in many types as co-expression, shared functional annotations, co-occurrence in literature and co-regulation [29,53-55]. These highly heterogeneous data contributed not only to inferring stronger relationships through the accumulation of evidence, but also providing broader coverage than any single data source.

Finally, MAXCOM could potentially be applied to find combinatorial protein targets and then help design network drugs. Here a disease is considered as the perturbations of the complex intracellular and intercellular network that links tissue and organ systems [56]. The ability of exploring molecular complexity of a particular disease at protein complex level will lead to the identification of the molecular relationships among distinct phenotypes. Thus, systematically predicting and analyzing disease-associated protein complexes could be useful for investigation of mechanisms underlying diseases, and could help to identify combinational drug targets and biomarkers.

## Competing interests

The authors declare that they have no competing interests.

## Authors' contributions

RJ provided guidance and planning for the project. YC produced the program and wrote the manuscript, particularly producing the results section. YC, TJ and SZ contributed in preparing data and analysis of the results. All authors read and approved the final manuscript.

## Declarations

Publication of this article was funded by the corresponding author.

This article has been published as part of *BMC Systems Biology *Volume 8 Supplement 1, 2014: Selected articles from the Twelfth Asia Pacific Bioinformatics Conference (APBC 2014): Systems Biology. The full contents of the supplement are available online at http://www.biomedcentral.com/bmcsystbiol/supplements/8/S1.

## References

[B1] Schadt EE Molecular networks as sensors and drivers of common human diseases Nature 2009 461 7261 218 223 10.1038/nature08454 19741703

[B2] Zhao J Lee SH Huss M Holme P The network organization of cancer-associated protein complexes in human tissues Scientific reports 2013 3 1583 23567845 10.1038/srep01583 PMC3620901

[B3] Chairerg P Tantavisut S Tanavalee A Tuangjaruwinai W Panchaprateep R Asawanonda P Cast application of four weeks' duration significantly affects hair length, diameter and density The Journal of dermatological treatment 2013 10.3109/09546634.2013.789471 23550688

[B4] Jiang R Yang H Sun F Chen T Searching for interpretable rules for disease mutations: a simulated annealing bump hunting strategy BMC bioinformatics 2006 7 417 10.1186/1471-2105-7-417 16984653 PMC1618409

[B5] Jiang R Yang H Zhou L Kuo CC Sun F Chen T Sequence-based prioritization of nonsynonymous single-nucleotide polymorphisms for the study of disease mutations American journal of human genetics 2007 81 2 346 360 10.1086/519747 17668383 PMC1950793

[B6] Frazer KA Ballinger DG Cox DR Hinds DA Stuve LL Gibbs RA Belmont JW Boudreau A Hardenbol P Leal SM A second generation human haplotype map of over 3.1 million SNPs Nature 2007 449 7164 851 861 10.1038/nature06258 17943122 PMC2689609

[B7] Hindorff LA Sethupathy P Junkins HA Ramos EM Mehta JP Collins FS Manolio TA Potential etiologic and functional implications of genome-wide association loci for human diseases and traits Proceedings of the National Academy of Sciences of the United States of America 2009 106 23 9362 9367 10.1073/pnas.0903103106 19474294 PMC2687147

[B8] Tang W Wu X Jiang R Li Y Epistatic module detection for case-control studies: a Bayesian model with a Gibbs sampling strategy PLoS genetics 2009 5 5 e1000464 10.1371/journal.pgen.1000464 19412524 PMC2669883

[B9] Jiang R Tang W Wu X Fu W A random forest approach to the detection of epistatic interactions in case-control studies BMC bioinformatics 2009 10 Suppl 1 S65 10.1186/1471-2105-10-S1-S65 19208169 PMC2648748

[B10] Calin GA Croce CM MicroRNA signatures in human cancers Nature reviews Cancer 2006 6 11 857 866 10.1038/nrc1997 17060945

[B11] Cheetham SW Gruhl F Mattick JS Dinger ME Long noncoding RNAs and the genetics of cancer British journal of cancer 2013 108 12 2419 2425 10.1038/bjc.2013.233 23660942 PMC3694235

[B12] Moreau Y Tranchevent LC Computational tools for prioritizing candidate genes: boosting disease gene discovery Nature reviews Genetics 2012 13 8 523 536 10.1038/nrg3253 22751426

[B13] Portela A Esteller M Epigenetic modifications and human disease Nature biotechnology 2010 28 10 1057 1068 10.1038/nbt.1685 20944598

[B14] Santen GW Aten E Sun Y Almomani R Gilissen C Nielsen M Kant SG Snoeck IN Peeters EA Hilhorst-Hofstee Y Mutations in SWI/SNF chromatin remodeling complex gene ARID1B cause Coffin-Siris syndrome Nature genetics 2012 44 4 379 380 10.1038/ng.2217 22426309

[B15] Tsurusaki Y Okamoto N Ohashi H Kosho T Imai Y Hibi-Ko Y Kaname T Naritomi K Kawame H Wakui K Mutations affecting components of the SWI/SNF complex cause Coffin-Siris syndrome Nature genetics 2012 44 4 376 378 10.1038/ng.2219 22426308

[B16] Van Houdt JK Nowakowska BA Sousa SB van Schaik BD Seuntjens E Avonce N Sifrim A Abdul-Rahman OA van den Boogaard MJ Bottani A Heterozygous missense mutations in SMARCA2 cause Nicolaides-Baraitser syndrome Nature genetics 2012 44 4 445 449 S441 10.1038/ng.1105 22366787

[B17] Wilson BG Roberts CW SWI/SNF nucleosome remodellers and cancer Nature reviews Cancer 2011 11 7 481 492 10.1038/nrc3068 21654818

[B18] Kadoch C Hargreaves DC Hodges C Elias L Ho L Ranish J Crabtree GR Proteomic and bioinformatic analysis of mammalian SWI/SNF complexes identifies extensive roles in human malignancy Nature genetics 2013 45 6 592 601 10.1038/ng.2628 23644491 PMC3667980

[B19] Santidrian AF Matsuno-Yagi A Ritland M Seo BB LeBoeuf SE Gay LJ Yagi T Felding-Habermann B Mitochondrial complex I activity and NAD+/NADH balance regulate breast cancer progression The Journal of clinical investigation 2013 123 3 1068 1081 10.1172/JCI64264 23426180 PMC3582128

[B20] Kalaitzidis D Sykes SM Wang Z Punt N Tang Y Ragu C Sinha AU Lane SW Souza AL Clish CB mTOR complex 1 plays critical roles in hematopoiesis and Pten-loss-evoked leukemogenesis Cell stem cell 2012 11 3 429 439 10.1016/j.stem.2012.06.009 22958934 PMC3743253

[B21] Krogan NJ Cagney G Yu H Zhong G Guo X Ignatchenko A Li J Pu S Datta N Tikuisis AP Global landscape of protein complexes in the yeast Saccharomyces cerevisiae Nature 2006 440 7084 637 643 10.1038/nature04670 16554755

[B22] Babu M Vlasblom J Pu S Guo X Graham C Bean BD Burston HE Vizeacoumar FJ Snider J Phanse S Interaction landscape of membrane-protein complexes in Saccharomyces cerevisiae Nature 2012 489 7417 585 589 10.1038/nature11354 22940862

[B23] Michaut M Baryshnikova A Costanzo M Myers CL Andrews BJ Boone C Bader GD Protein complexes are central in the yeast genetic landscape PLoS computational biology 2011 7 2 e1001092 10.1371/journal.pcbi.1001092 21390331 PMC3044758

[B24] Guruharsha KG Rual JF Zhai B Mintseris J Vaidya P Vaidya N Beekman C Wong C Rhee DY Cenaj O A protein complex network of Drosophila melanogaster Cell 2011 147 3 690 703 10.1016/j.cell.2011.08.047 22036573 PMC3319048

[B25] Kikugawa S Nishikata K Murakami K Sato Y Suzuki M Altaf-Ul-Amin M Kanaya S Imanishi T PCDq: human protein complex database with quality index which summarizes different levels of evidences of protein complexes predicted from h-invitational protein-protein interactions integrative dataset BMC systems biology 2012 6 Suppl 2 S7 10.1186/1752-0509-6-S2-S7 23282181 PMC3521179

[B26] Lage K Karlberg EO Storling ZM Olason PI Pedersen AG Rigina O Hinsby AM Tumer Z Pociot F Tommerup N A human phenome-interactome network of protein complexes implicated in genetic disorders Nature biotechnology 2007 25 3 309 316 10.1038/nbt1295 17344885

[B27] Vanunu O Magger O Ruppin E Shlomi T Sharan R Associating genes and protein complexes with disease via network propagation PLoS computational biology 2010 6 1 e1000641 10.1371/journal.pcbi.1000641 20090828 PMC2797085

[B28] Yang P Li X Wu M Kwoh CK Ng SK Inferring gene-phenotype associations via global protein complex network propagation PLoS One 2011 6 7 e21502 10.1371/journal.pone.0021502 21799737 PMC3143124

[B29] Aerts S Lambrechts D Maity S Van Loo P Coessens B De Smet F Tranchevent LC De Moor B Marynen P Hassan B Gene prioritization through genomic data fusion Nature biotechnology 2006 24 5 537 544 10.1038/nbt1203 16680138

[B30] Wu X Jiang R Zhang MQ Li S Network-based global inference of human disease genes Molecular systems biology 2008 4 189 18463613 10.1038/msb.2008.27 PMC2424293

[B31] Wu X Liu Q Jiang R Align human interactome with phenome to identify causative genes and networks underlying disease families Bioinformatics 2009 25 1 98 104 10.1093/bioinformatics/btn593 19010805

[B32] Wang W Zhang W Jiang R Luan Y Prioritisation of associations between protein domains and complex diseases using domain-domain interaction networks IET systems biology 2010 4 3 212 222 10.1049/iet-syb.2009.0037 20500001

[B33] Chen Y Jiang T Jiang R Uncover disease genes by maximizing information flow in the phenome-interactome network Bioinformatics 2011 27 13 i167 176 10.1093/bioinformatics/btr213 21685067 PMC3117332

[B34] Zhang W Sun F Jiang R Integrating multiple protein-protein interaction networks to prioritize disease genes: a Bayesian regression approach BMC bioinformatics 2011 12 Suppl 1 S11 10.1186/1471-2105-12-S1-S11 21342540 PMC3044265

[B35] Zhang W Chen Y Sun F Jiang R DomainRBF: a Bayesian regression approach to the prioritization of candidate domains for complex diseases BMC systems biology 2011 5 55 10.1186/1752-0509-5-55 21504591 PMC3108930

[B36] Jiang R Gan M He P Constructing a gene semantic similarity network for the inference of disease genes BMC systems biology 2011 5 Suppl 2 S2 10.1186/1752-0509-5-S2-S2 22784573 PMC3287482

[B37] van Driel MA Bruggeman J Vriend G Brunner HG Leunissen JA A text-mining analysis of the human phenome European journal of human genetics: EJHG 2006 14 5 535 542 10.1038/sj.ejhg.5201585 16493445

[B38] Keshava Prasad TS Goel R Kandasamy K Keerthikumar S Kumar S Mathivanan S Telikicherla D Raju R Shafreen B Venugopal A Human Protein Reference Database--2009 update Nucleic acids research 2009 37 Database D767 772 10.1093/nar/gkn892 18988627 PMC2686490

[B39] Smedley D Haider S Ballester B Holland R London D Thorisson G Kasprzyk A BioMart--biological queries made easy BMC genomics 2009 10 22 10.1186/1471-2164-10-22 19144180 PMC2649164

[B40] Goldberg AV Rao S Beyond the flow decomposition barrier Journal of the ACM (JACM) 1998 45 5 783 797 10.1145/290179.290181

[B41] Ruepp A Waegele B Lechner M Brauner B Dunger-Kaltenbach I Fobo G Frishman G Montrone C Mewes HW CORUM: the comprehensive resource of mammalian protein complexes--2009 Nucleic acids research 2010 38 Database D497 501 10.1093/nar/gkp914 19884131 PMC2808912

[B42] Pilpel Y Noise in biological systems: pros, cons, and mechanisms of control Methods Mol Biol 2011 759 407 425 10.1007/978-1-61779-173-4_23 21863500

[B43] Ladbury JE Arold ST Noise in cellular signaling pathways: causes and effects Trends in biochemical sciences 2012 37 5 173 178 10.1016/j.tibs.2012.01.001 22341496 PMC3348409

[B44] Hamosh A Scott AF Amberger JS Bocchini CA McKusick VA Online Mendelian Inheritance in Man (OMIM), a knowledgebase of human genes and genetic disorders Nucleic acids research 2005 33 Database D514 517 15608251 10.1093/nar/gki033 PMC539987

[B45] Huang da W Sherman BT Lempicki RA Systematic and integrative analysis of large gene lists using DAVID bioinformatics resources Nature protocols 2009 4 1 44 57 19131956 10.1038/nprot.2008.211

[B46] Huang da W Sherman BT Lempicki RA Bioinformatics enrichment tools: paths toward the comprehensive functional analysis of large gene lists Nucleic acids research 2009 37 1 1 13 10.1093/nar/gkn923 19033363 PMC2615629

[B47] Roberts CW Orkin SH The SWI/SNF complex--chromatin and cancer Nature reviews Cancer 2004 4 2 133 142 10.1038/nrc1273 14964309

[B48] Euskirchen G Auerbach RK Snyder M SWI/SNF chromatin-remodeling factors: multiscale analyses and diverse functions The Journal of biological chemistry 2012 287 37 30897 30905 10.1074/jbc.R111.309302 22952240 PMC3438922

[B49] Ong CT Corces VG Enhancer function: new insights into the regulation of tissue-specific gene expression Nature reviews Genetics 2011 12 4 283 293 10.1038/nrg2957 21358745 PMC3175006

[B50] Wan J Oliver VF Zhu H Zack DJ Qian J Merbs SL Integrative analysis of tissue-specific methylation and alternative splicing identifies conserved transcription factor binding motifs Nucleic acids research 2013 10.1093/nar/gkt652 PMC3794605 23887936

[B51] Ellis JD Barrios-Rodiles M Colak R Irimia M Kim T Calarco JA Wang X Pan Q O'Hanlon D Kim PM Tissue-specific alternative splicing remodels protein-protein interaction networks Molecular cell 2012 46 6 884 892 10.1016/j.molcel.2012.05.037 22749401

[B52] Zhong Q Simonis N Li QR Charloteaux B Heuze F Klitgord N Tam S Yu H Venkatesan K Mou D Edgetic perturbation models of human inherited disorders Molecular systems biology 2009 5 321 19888216 10.1038/msb.2009.80 PMC2795474

[B53] Stuart JM Segal E Koller D Kim SK A gene-coexpression network for global discovery of conserved genetic modules Science 2003 302 5643 249 255 10.1126/science.1087447 12934013

[B54] Ma X Lee H Wang L Sun F CGI: a new approach for prioritizing genes by combining gene expression and protein-protein interaction data Bioinformatics 2007 23 2 215 221 10.1093/bioinformatics/btl569 17098772

[B55] Jenssen TK Laegreid A Komorowski J Hovig E A literature network of human genes for high-throughput analysis of gene expression Nature genetics 2001 28 1 21 28 11326270 10.1038/ng0501-21

[B56] Barabasi AL Gulbahce N Loscalzo J Network medicine: a network-based approach to human disease Nature reviews Genetics 2011 12 1 56 68 10.1038/nrg2918 21164525 PMC3140052

